# Coexisting DCIS and phyllodes breast tumors in Young Chinese women: Case series

**DOI:** 10.1016/j.ijscr.2019.01.045

**Published:** 2019-02-08

**Authors:** Luona Sun, Roger Zhu, Paula Ginter, Manmeet Malik, Kap-Jae Sung, J Melissa Hughes, Beth Siegel, Jacqueline Tsai

**Affiliations:** a Department of Surgery, New York Presbyterian Queens, United States; b Department of Pathology, New York Presbyterian Queens, United States

## Abstract

**Introduction:**

Breast cystosarcoma phyllodes tumors are rare and can be benign or malignant. All sub-divisions of phyllodes tumor—benign, borderline and malignant, can harbor carcinomas, although the incidence is extremely rare. Methods: We present two nonconsecutive cases of coexisting ductal carcinoma in situ (DCIS) and phyllodes breast tumors in young patients.

**Methods & Case Presentation:**

Retrospective review of two patient’s medical record was performed.

**Case 1:**

30-year-old female underwent excisional biopsy for 3.48 cm mass found on ultrasound. Pathology revealed malignant phyllodes tumor with positive margin. On re-excision, patient was found to have 1.5 cm area of ductal carcinoma in situ (DCIS) with positive margin. Patient then underwent re-reexicision of DCIS with negative margin. Patient underwent chemotherapy and tamoxifen for three years without evidence of disease.

**Case 2:**

30-year-old female presented with 1.3 cm lesion found on ultrasound which core needle biopsy revealed a fibroepithelial tumor. Patient subsequently underwent excision biopsy which found 1.5 cm benign phyllodes tumor and 3.5 mm DCIS within the phyllodes tumor with negative margins. Patient declined additional chemotherapy or hormonal therapy and is currently considering mastectomy.

**Conclusion:**

Phyllodes tumors are rare and ones with a coexisting carcinoma are even less frequently encountered. The treatment plan can change upon diagnosis of the carcinoma via the pathology. Treatment should be guided by the type and stage of carcinoma detected which may include additional surgical resection and lymph node sampling.

## Highlights

• Breast cystosarcomaphyllodes tumors are rare and can be benign or malignant.
• These tumors can harbor carcinomas, although the incidence is extremely rare.
• Although challenging, the detection of a carcinoma component in phyllodes tumor is important, as it can dictate the need for lymph node sampling and possible adjuvant therapies such as radiation and systemic management.

## 1 Introduction

Breast cystosarcoma phyllodes tumors are rare and can be benign or malignant. Despite the malignant potential, they comprise less than 1% of all primary breast tumors and 2–3% of the fibroepithelial subcategory [1,2]. All sub-divisions of phyllodes tumor—benign, borderline and malignant, can harbor carcinomas, although the incidence is extremely rare [2]. We present two cases of coexisting ductal carcinoma in situ (DCIS) and phyllodes breast tumors in young patients in a community hospital.

## 2 Methods & case presentation

Retrospective review of the two identified patients’ medical record including laboratory values, pathology reports, and imaging, were performed. Current case report is in line with the PROCESS criteria [3].

## 3 Case 1

**Fig. 1:**
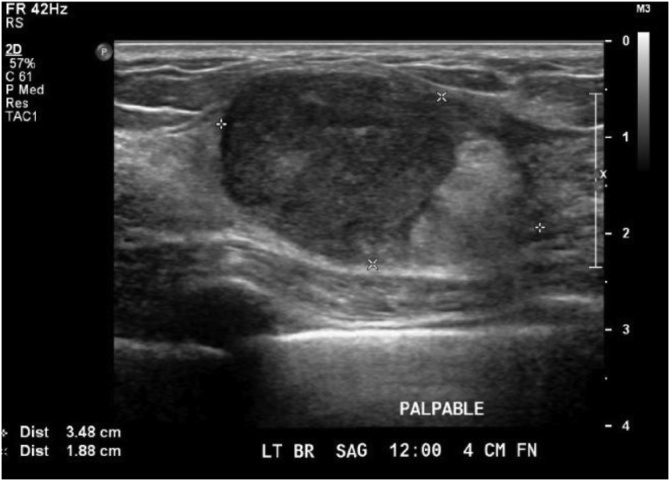
Ultrasound of left breast showing an avascular heterogeneous mass.

A 30-year-old Chinese woman, G1P1, with a history of an excisional biopsy for a benign right breast mass seven years ago, presented with a new palpable left breast mass in the postpartum period. She was not breastfeeding and had no family history of breast or ovarian cancer. On clinical breast exam, there was a 3.6 cm mass at the 12 o’clock position of the left breast, 4 cm from the nipple, with a normal axillary examination. On ultrasound, there was a corresponding heterogenous 3.48 cm mass (Fig. 1).

**Fig. 2:**
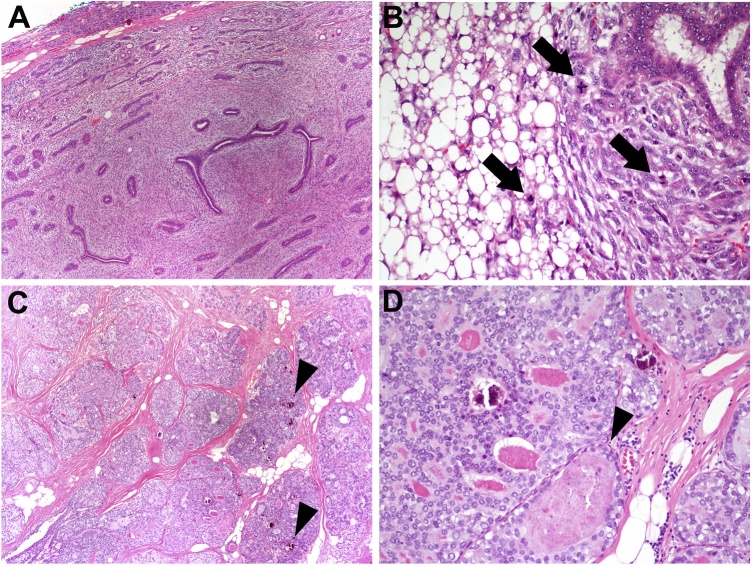
Malignant phyllodes tumor with ductal carcinoma in situ (DCIS) in surrounding tissue. (A) Low-power magnification image shows a well-circumscribed phyllodes tumor with moderately cellular stroma. (B) Some areas of the phyllodes tumor demonstrated increased periductal stromal cellularity (right) with numerous mitoses (arrows), including atypical forms, and malignant liposarcomatous transformation (left). The stromal cells demonstrate marked cytologic atypia. (C) The subsequent re-excision specimen shows expanded ducts involved by DCIS with solid and cribriform architectural growth and calcifications (arrowheads). (D) Higher magnification of DCIS shows a monotonous population of ductal epithelium with intermediate grade nuclei and focal necrosis (arrowhead). The final re-excision demonstrated similar DCIS with calcifications (not shown).

Ultrasound-guided core needle biopsy demonstrated benign breast tissue with focal secretary changes and chronic inflammation. The pathology was considered to be discordant with imaging findings. The patient underwent a left breast excisional biopsy which revealed a 3.2 cm malignant phyllodes tumor focally extending to inferior, medial, and posterior margins with noted tumor <1 mm from all other margins. Given the close and positive margins, the patient underwent a re-excision of all margins to achieve a final 1 cm in all margins. Pathology demonstrated no further evidence of the malignant phyllodes tumor, however, incidentally noted a 1.5 cm area of DCIS at the lateral margin, ER+ (90%) and PR- (negative) with a positive margin (Fig. 2).

Bilateral diagnostic mammogram demonstrated a small cluster of punctate calcifications in the left upper outer quadrant, in the posterior aspect of the lumpectomy cavity which was suspicious for malignancy. The patient was referred for genetic counseling and testing which was negative for BRAC 1/2. She subsequently underwent a left breast re-excision lumpectomy of the lateral margin along with wire localization excisional biopsy of the calcifications. Final pathology revealed an additional 7 mm of intermediate grade DCIS, with no evidence of malignant phyllodes tumor and a negative final margin. She completed adjuvant radiation therapy and was placed on tamoxifen. Follow-up mammography, ultrasound, and clinical exam had been stable with no evidence of new or recurrent malignancy for almost three years.

## 4 Case 2

**Fig. 3:**
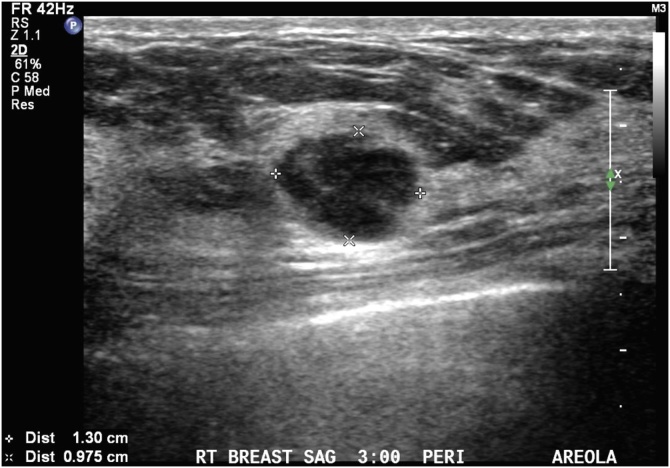
Ultrasound of right breast showing a heterogeneous, hypoechoic nodule.

**Fig. 4:**
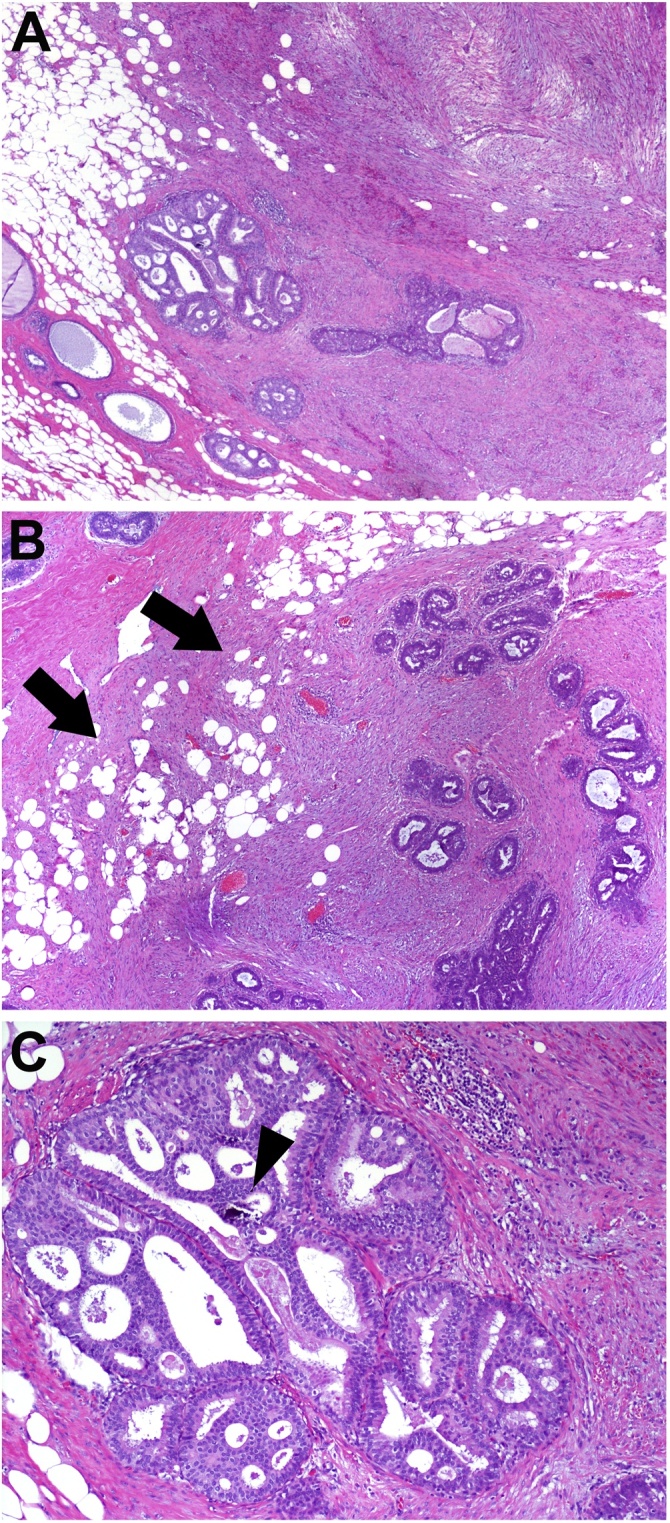
Ductal carcinoma in situ (DCIS) within a benign phyllodes tumor. (A) Low-magnification image shows a phyllodes tumor with cellular and expanded stroma (upper right) and ducts expanded by proliferating epithelium (left = DCIS, right = usual ductal hyperplasia). (B) The phyllodes tumor demonstrates infiltrative edges, extending into adjacent adipose tissue (arrows). Only mild stromal atypia was identified. Mitoses were infrequent. (C) Higher magnification of the DCIS demonstrates a monotonous population of epithelial cells with intermediate grade nuclei and rigid cribriform architecture. Minute calcifications are associated with DCIS (arrowhead).

A 30-year-old nulliparous Chinese woman presented with a palpable right breast mass for one month. On clinical exam, a 1 cm firm nodule was palpated in the medial aspect of the right breast. On the diagnostic US, there was a 1.3 cm heterogeneous, hypoechoic nodule at the 3 o’clock position in the periareolar region with no suspicious microcalcifications or architectural distortion (Fig. 3). Subsequent ultrasound-guided core needle biopsy demonstrated a fibroepithelial tumor for which the patient underwent an excisional biopsy. Pathology revealed a 1.5 cm benign phyllodes tumor with mild cytologic atypia and no stromal overgrowth; additionally, a 3.5 mm intermediate grade DCIS was found as a single focus within the benign phyllodes tumor, ER+ (85%) and PR+ (95%) (Fig. 4). DCIS was 2 mm from the anterior margin and >5 mm for all other margins. Phyllodes tumor was <1 mm from anterior and posterior margins, at 1 mm from the medial margin and >5 mm from remaining margins. She underwent genetic counseling and testing which was negative for BRCA 1/2. The patient desired to start a family and declined radiation and tamoxifen treatment. At the last follow up at 1 month after surgery, she was considering bilateral nipple-sparing mastectomy.

## 5 Discussion

Breast cystosarcoma phyllodes tumors are rare and comprise less than 1% of all primary breast tumors and 2–3% of the fibroepithelial subcategory [1,2]. They are fibroepithelial tumors with epithelial and cellular stromal content. Cystosarcoma phyllodes tumor was named after its architectural features on histology: the elongated epithelial linings giving the name cystosarcoma versus the leaf-like structure of the hyperplastic stroma giving the name of phyllodes [7,11].

Some key histological factors are required to make the diagnosis of phyllodes tumors. Factors such as stromal overgrowth, hypercellularity, atypia, high mitosis rate, infiltrative borders and the presence of necrosis [1,2,4,6], are necessary to distinguish the tumor between benign and malignant phyllodes. Although, they have a structural similarity with its benign relative--the fibroadenoma, cystosarcoma phyllodes have more significant hypercellularity and overgrowth of the stromal protrusion. The stromal hyperplasia may comprise various densities and pleomorphic cells with some mitotic figures. Clinically, the suspicion should be raised if a breast tumor has a large size and a rapid growth rate [9]. A combination of features from microscopic review and clinical exam will lead to the final diagnosis of cystosarcoma phyllodes tumors.

Phyllodes tumors are further divided into three groups of benign, borderline or malignant tumors according to the World Health Organization (WHO) 2012 classification [1,2,4,[6], [7], [8],^12^]. The diagnostic criteria are also based on similar criteria for the initial diagnosis: stromal overgrowth, hypercellularity, atypia, high mitosis rate, infiltrative borders and the presence of necrosis. The malignancy potential is based on a large tumor size (>4 cm in diameter), the loss of defined margins and more infiltrative borders, more cellular atypia and increased mitotic activity [3,11]. More detailed diagnostic differentiation is used for this subdivision. For example, a mitotic rate of less than 5 per 10 high power field (HPF) suggests “benign” subdivision, between 5–10 HPF “borderline”, and more than 10 per 10 HPF “malignancy” [6]. In the literature, the histologic distribution of phyllodes tumors into benign, borderline, and malignant is 54–58 percent, 12 percent, and 30–33 percent, respectively [14,15].

When a coexisting carcinoma arises, the distinction becomes challenging [2,7,11]. Although extremely rare, all sub-divisions of phyllodes tumorcan harbor carcinomas. These carcinomas can be carried in two forms; either present as a separate lesion in the ipsilateral or contralateral breast; or inhabiting within the phyllodes tumor itself [[4], [5], [6], [7]]. Different kinds of carcinomas were reported in the literature: DCIS, tubular carcinoma, invasive ductal /lobular carcinoma, and squamous cell carcinoma [6,8]. One diagnostic challenge of coexisting carcinoma with phyllodes is to differentiate from the carcinosarcoma—a subtype of metaplastic carcinoma. By WHO classification, the differentiation of carcinosarcoma is the presence of both epithelial and mesenchymal components, with mesenchymal component showing malignant microscopic features without epithelial makers. Nonetheless, it remains a diagnostic challenge [2,7,11].

Phyllodes tumors occur within a wide age distribution ranging from the 2nd to the 6th decade of life [1,2]. Patients who have coexisting carcinoma with phyllodes are more likely to be older than 50 years of age [4,6]. However, there have been several reported cases of young patients in their 20–30 s with coexisting DCIS. Our reported patient cases follow a similar young age of diagnosis. Additionally, the age distribution follows a pattern of ethnic predilection, with Asian females with phyllodes tumors diagnosed at a younger age compared to Caucasian females [1,2,8,11]. In the Bernstein et al study done in Los Angeles in1993, Asian and Latina's patients were significantly younger than non-Latina white patients. Similarly, in the Panda et al study in 2016, phyllodes tumors found in Asian countries occurred primarily in the 25–30 year age group and accounted for a higher proportion of primary breast tumors. Both of our patients were born in China and came to the US at a later age. The exact genetic and environmental contributing factors to this variation are unknown.

Surgery has been viewed as the primary treatment for phyllodes tumors [2,6,7]. The options are a lumpectomy versus a mastectomy. The main determining factor is the size of the mass; the breast size and the acceptable cosmetic outcome. Historically, the average diameter of phyllodes is 4–5 cm [8]. As in our case series, the lesions were 3.48 cm and 1.5 cm. Due to the small size, both of our patients underwent lumpectomy. In Chaney et al study in 2000, the median phyllodes tumor size of the patients who underwent lumpectomy was 4 cm, while patients who underwent mastectomy had a median tumor size of 10 cm. No relation between tumor size and the grade of histology was observed [2].

Once the surgical choice has been made, the question of margin arises. Wide local excision of the tumor with negative margins results in 90% local control rate and is recommended when cosmetic appearance is acceptable [2]. However, the exact margin length is controversial. The current National Comprehensive Cancer Network (NCCN) guidelines for the management of phyllodes tumors recommends wide excision with margins ≥1 cm and recommends against axillary staging [9,10]. However, in recent years, a smaller margin for benign phyllodes tumors has been discussed and has started to gain popularity. In Moutte et al study in 2016, 77 patients were reviewed and identified small negative surgical margins: <10 mm in 89%. Re-excision was not performed and there was no increase in the local recurrence rate (4%) observed when compared to recurrence rates over 58 months follow up. Thus, they recommended against re-excision for benign phyllodes with close or positive surgical margins to achieve margins beyond 1 cm. Regardless of the margin, regular clinical and imaging follow-up is highly recommended, as most recurrences happen during the first two years of the initial surgery.

Although challenging, the detection of a carcinoma component in phyllodes tumor is important, as it can dictate the need for lymph node sampling and possible adjuvant therapies such as radiation and systemic management [6]. The current NCCN guideline recommends against lymph node sampling for isolated phyllodes, as malignant phyllodes tumors more commonly spread via a hematogenous route and lymphatic metastases are extremely rare [6,7,13]. In the case of a coexisting carcinoma, which is often diagnosed only after the excision, the treatment plan can change [[6], [7], [8], [9],^14^].

Routine use of radiation is not recommended for phyllodes tumors as there are no randomized studies supporting the use of post-operative radiation. An exception is when local recurrence would result in significant morbidity at which point radiation therapy is considered following a sarcoma treatment protocol [9].

Despite the generally favorable prognosis of phyllodes,13–20% of patients with malignant phyllodes experience distant metastasis within10 years. No standard chemotherapy is currently recommended [6,7]. Malignant phyllodes tumors tend to be more aggressive and have a higher chance of metastasis, however, metastases have been reported in up to 8% of initially histologically benign tumors in a 10-year period [6]. Although stromal overgrowth is the strongest predictor of distant metastasis, it is hard to predict. Local failure rates for benign tumors range between 5–15% and 20–30% for malignant tumors [2,7].

## 6 Conclusion

Phyllodes tumors are rare and ones with a coexisting carcinoma are even less frequently encountered. The treatment plan can change upon diagnosis of the carcinoma via the pathology. Treatment should be guided by the type and stage of carcinoma detected which may include additional surgical resection and lymph node sampling.

## Conflicts of interest

N/A.

## Sources of funding

N/A.

## Ethical approval

The NYP/Queens Institutional Review Board (IRB) has reviewed and determined that the proposed project does not meet definition of research involving human subjects as per federal regulation (45CFR46.102); therefore, it may be conducted without further IRB review.

## Consent

Written informed consent was obtained from the patients for publication of this case series and accompanying images. A copy of the written consent is available for review by the Editor-in-Chief of this journal on request

## Author contribution

Luona Sun – Preparation of Manuscript

Roger Zhu – Preparation of Case Presentation and Edit of manuscript

Paula Ginter – Preparation of histology imagings

Manmeet Malik – Review and edit of manuscript

Kap-Jae Sung – Review and edit of manuscript

Melissa Hughes – Review and edit of manuscript

Beth Siegel – Review and edit of manuscript

Jacqueline Tsai – Review and edit of manuscript

## Registration of research studies

researchregistry4509

Case Series.

## Guarantor

Luona Sun, MD.

Provenance and peer review

Not commissioned, externally peer-reviewed

## Credit author statement

Below are the authors who made significant contribution to the completion of the manuscript.

Luona Sun- Preparation of Manuscript

Roger Zhu- Preparation of Case Presentation and Edit of manuscript

Paula Ginter – Preparation of histology imagings

Manmeet Malik – Review and edit of manuscript

Kap-Jae Sung - Review and edit of manuscript

Melissa Hughes - Review and edit of manuscript

Beth Siegel - Review and edit of manuscript

Jacqueline Tsai - Review and edit of manuscript
